# Multi-model assessment identifies livestock grazing as a major contributor to variation in European Union land and water footprints

**DOI:** 10.1038/s43016-023-00797-8

**Published:** 2023-07-17

**Authors:** Davy Vanham, Martin Bruckner, Florian Schwarzmueller, Joep Schyns, Thomas Kastner

**Affiliations:** 1 European Commission, Joint Research Centre (JRC), Ispra, Italy; 2 Institute for Ecological Economics, Vienna University of Economics and Business (WU), Vienna, Austria; 3 Institute of Environmental Engineering, ETH Zurich, Zurich, Switzerland; 4 Senckenberg Biodiversity and Climate Research Centre (SBiK-F), Frankfurt am Main, Germany; 5 Multidisciplinary Water Management Group, Faculty of Engineering Technology, University of Twente, Enschede, the Netherlands

**Keywords:** Water resources, Agriculture

## Abstract

Food systems are the largest users of land and water resources worldwide. Using a multi-model approach to track food through the global trade network, we calculated the land footprint (LF) and water footprint (WF) of food consumption in the European Union (EU). We estimated the EU LF as 140–222 Mha yr^−1^ and WF as 569–918 km^3^ yr^−1^. These amounts are 5–7% of the global LF and 6–10% of the global WF of agriculture, with the EU representing 6% of the global population. We also calculated the global LF of livestock grazing, accounting only for grass eaten, to be 1,411–1,657 Mha yr^−1^, and the global LF of agriculture to be 2,809–3,014 Mha yr^−1^, which is about two-thirds of what the Food and Agriculture Organization Statistics (FAOSTAT) database reports. We discuss here the different methods for calculating the LF for livestock grazing, underscoring the need for a consistent methodology when monitoring the food LF and WF reduction goals set by the EU’s Farm To Fork Strategy.

## Abstract (web summary)

Reducing the environmental pressure and impact of food production is central to the European Union’s Farm to Fork Strategy. This study applies a multi-model approach to track food through the global trade network, estimating the land and water footprints of food consumption in the 27 member states of the European Union.

## Main

The food system is a major contributor to different environmental pressures, such as water and land use, and impacts, including land-use change and water stress^1,2^. Accounting for the land and water resources used by the food system is key to defining sustainable food system policies. The European Green Deal and its Farm to Fork Strategy^3^ aim at a sustainable food system along the whole value chain, from primary production to final consumption^4^. The quantification of the land footprint (LF) and the water footprint (WF) of food consumption in the European Union (EU), and the setting of reduction targets, are key topics for this strategy.

Environmental footprints can be calculated using different methodological approaches^5–7^, yielding different results for the same geographical region^8–11^. These methods range from process-based approaches to environmentally extended multi-regional input–output (EE-MRIO) approaches^6^. In addition, specific calculation assumptions can yield very different results. While it is common in WF assessments to only account for grass eaten by grazing livestock^12^, land-use accounting often attributes all grazing land to the LF of the livestock^13^.

In recent years, EE-MRIO models have been widely used to study the physical flows of the materials induced by production and consumption activities in the global economy^14^. However, the robustness of MRIO-based calculations of global physical biomass flows has been questioned due to three main problematic areas^15^. First, the monetary structure of the economy that underlies the basis of MRIO models does not always represent the quantities of physical product flows correctly. Due to price variations of product flows between different customers, the assumption of proportionality between monetary and physical flows can lead to over- or underestimations^11^. Second, the limited detail of monetary input–output tables results in a grouping of diverse products into homogeneous sectors^16^. Third, there are discrepancies between agricultural and forestry statistics reported in physical units on the one hand, and macroeconomic production statistics in monetary units on the other, for example, due to different system boundaries^17^. To reduce uncertainties arising from the above-mentioned limitations of input–output models, a number of studies have suggested moving from sector-level economic data towards more detailed physical data^14,15^.

In this study, we first applied a multi-model approach to track the EU WF and LF through the global trade network up to final consumption, and then used a harmonized approach for both the LF and WF of grazing. In particular, we used one physical trade model^18^ (PHYS) and three global MRIO models, that is, EXIOBASE^19^, FABIO^14^ and a hybrid model of both^20^ (HYBRID). PHYS is based on physical bilateral trade-flow data with an origin-tracing algorithm. It has been used in many studies^21^ and covers 191 primary agricultural products for 223 countries. EXIOBASE is a well-established and widely used, originally monetary, MRIO model now in its third version^19^. It covers 200 products and services, including 19 agricultural ones, for 44 countries and 5 aggregate regions (‘rest-of-the-world’ (ROW) Africa, ROW America, ROW Asia and Pacific, ROW Europe, and ROW Middle East). FABIO, or ‘Food and Agriculture Biomass Input–Output model’, is a relatively new MRIO model covering 130 commodities, of which 125 are agriculture and food products, for 191 countries and one ROW region. FABIO accounts for product flows in physical units. All the models incorporate the 27 EU member states (EU27) as different entities, which we combine under a single area for our results.

For each of the MRIO models, we used two different set-ups (EXIOBASE-min, EXIOBASE-max, FABIO-mass, FABIO-value, HYBRID-mass and HYBRID-value), resulting in a total of seven (including PHYS) model variations. For some of EXIOBASE’s 200 sectors, it is not completely clear whether the final consumption at the end of the supply chain incorporates food or not. An example is the ‘Real-estate services’ sector, where food might be served to personnel working in these services or to clients. At the same time, biofuels can be consumed for heat or transportation, bio-based detergents for cleaning, or fibres for textiles (Weinzettel and Wood^22^ have discussed this in detail). To illustrate the extent of this uncertainty, we distinguished between two extreme scenarios: in EXIOBASE-min, we drew the dividing line between food and non-food so as to only account for the footprint of those product groups in the ‘Food’ category whose main purpose is clearly the production of food (that is, agricultural and food industry products), while in EXIOBASE-max, we added all footprints of product groups and services to ‘Food’ that potentially include food (Supplementary Table 1). FABIO-mass and FABIO-value differ in that they were calculated using mass and value allocation, respectively, for by-products such as soybean oil and cake.

The harmonized approach to grazing follows the standard approach within the WF assessment, that is, accounting only for the grass eaten by livestock and not for the whole grazing area. The amounts of grass eaten were translated into an area-based estimate based on remotely sensed grassland productivity data.

We calculated the LF and WF of food consumption in all models for the current EU27, with harmonized input data for the year 2012, for a population of 441 million people^23^. To contextualize the results, we also computed the footprints for non-food uses of agricultural products. We estimated the LF and WF as pressure indicators, which quantify resource use along a supply chain^6,7^. The LF quantifies the use of cropland and grazing land resources, the WF the consumptive use of blue and green water resources^12^. Blue water refers to water in rivers, lakes, wetlands and aquifers, while green water is the soil water held in the unsaturated zone, originating from precipitation and eventually evaporating through and from plants and soils^24^. Irrigated agriculture receives blue water (from irrigation) as well as green water (from precipitation), while rainfed agriculture receives only green water. Apart from blue water for drinking and as service water, the WF of livestock comprises blue and green water in feed (both) and grazing (only green)^24,25^.

## LF and WF of EU food consumption

**Fig. 1:**
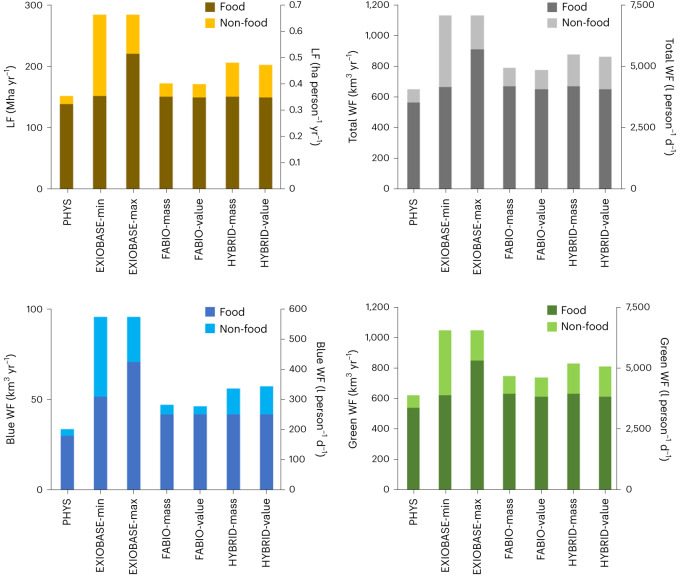
EU LF and WF for seven model variations. LF, total WF, blue WF and green WF for agricultural products consumed as ‘food’ and ‘non-food’ in the EU. All graphs are disaggregated between food and non-food components.

We calculated an EU food consumption LF ranging from 140.3 to 222.4 Mha yr^−^^1^ (or 0.318 to 0.504 ha person^−1^ yr^−1^; Fig. 1). The results of EXIOBASE-max stand out, with those of the other six model variations being very similar (140.3–152.9 Mha yr^−1^ or 0.318–0.347 ha person^−1^ yr^−1^).

The LF of agricultural products that are consumed as non-food products, which include, for example, biofuels or animal hides, is very high for the EXIOBASE model (133.3 and 63.7 Mha yr^−1^ for EXIOBASE-min and EXIOBASE-max, respectively), resulting in a total LF for these models of 286.1 Mha yr^−1^ (or 0.649 ha person^−1^ yr^−1^). Also, the LF of these non-food components is large for the HYBRID and FABIO models, ranging from 21.5 to 55.9 Mha yr^−1^. For PHYS, only 13.3 Mha yr^−1^ was computed.

The EU food consumption total WF (sum of the blue and green WF) was calculated to range from 569.3 km^3^ yr^−1^ (PHYS) to 917.8 km^3^ yr^−1^ (EXIOBASE-max; or 3,538 to 5,703 l person^−1^ d^−1^, Fig. 1). With the exception of EXIOBASE-max, the model variations provided similar results (569.3–674.3 km^3^ yr^−1^ or 3,538–4,190 l person^−1^ d^−1^).

Similar to the LF, the total WF of agricultural products consumed by the EU population as non-food products is very large for the EXIOBASE model (468.3 and 222.4 km^3^ yr^−1^ for EXIOBASE-min and EXIOBASE-max respectively), resulting in a total WF for these models of 1,140.2 km^3^ yr^−1^ (or 7,085 l person^−1^ d^−1^). The total WF of non-food components for the FABIO and HYBRID models ranges from 120.9 to 213.6 km^3^ yr^−1^ (or 795 to 1,327 l person^−1^ d^−1^). For PHYS, only 87 km^3^ yr^−1^ (or 540 l person^−1^ d^−1^) was computed.

The green WF, which represents the largest part of the total WF, follows the same pattern as the total WF across all the models.

The blue WF of food products, proportionately much smaller than the green WF, shows more variation between the different models, ranging from 29.7 km^3^ yr^−1^ (PHYS) to 70.0 km^3^ yr^−1^ (EXIOBASE-max; or 184 to 435 l person^−1^ d^−1^). For EXIOBASE-min, 50.9 km^3^ yr^−1^ (or 316 l person^−1^ d^−1^) was computed. For FABIO and HYBRID, the values are almost identical, that is, 41.2–41.4 km^3^ yr^−1^ (or 256–257 l person^−1^ d^−1^). PHYS shows the lowest value because water for food processing or livestock drinking water is presently not included in this model.

In line with the total WF, the blue WF of agricultural products consumed as non-food products is very high for the EXIOBASE model (43.8 and 24.8 km^3^ yr^−1^ for EXIOBASE-min and EXIOBASE-max, respectively), resulting in a blue WF for these models of 94.7 km^3^ yr^−1^ (or 589 l person^−1^ d^−1^). For HYBRID, the values are 13.9 and 15.4 km^3^ yr^−1^ (or 87 and 96 l person^−1^ d^−1^). HYBRID thus adds substantial value through non-food products in addition to food products. FABIO and PHYS show the lowest values of 4.8–5.1 km^3^ yr^−1^ (or 30–32 l person^−1^ d^−1^) and 3.7 km^3^ yr^−1^ (or 23 l person^−1^ d^−1^), respectively.

**Fig. 2:**
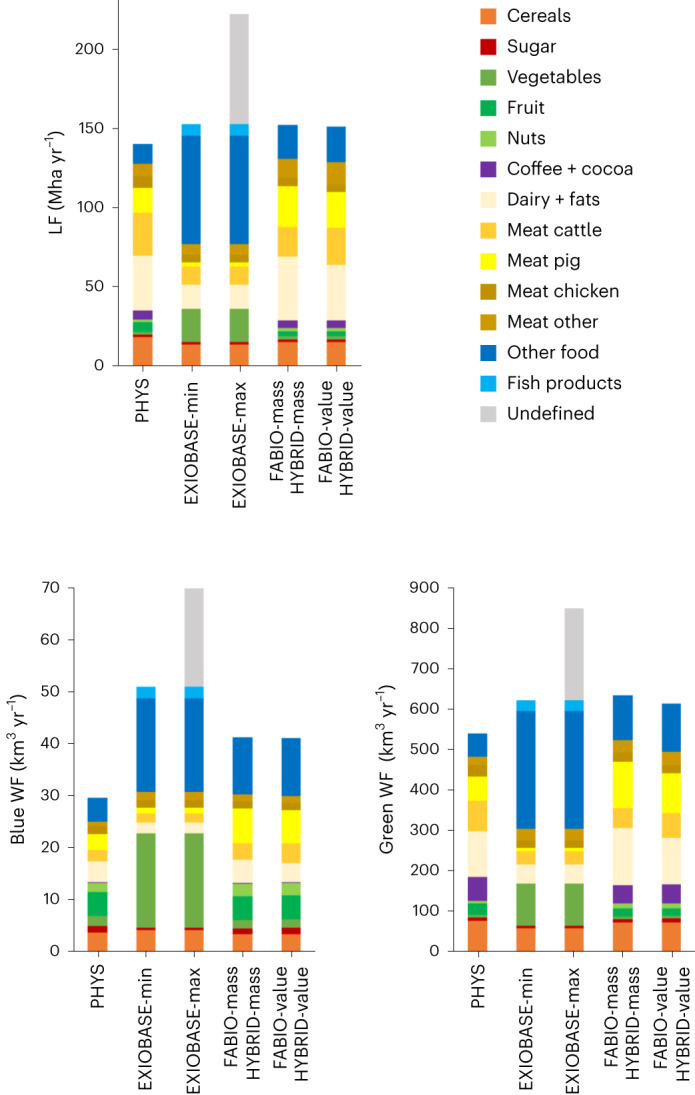
EU LF and WF per product group for the seven model variations. LF, blue WF and green WF values computed for different products in the seven models. In EXIOBASE, vegetables, fruit and nuts are one group. The product groups are defined in Supplementary Table 2. When non-food is not accounted for, FABIO-mass and HYBRID-mass become identical, as do FABIO-value and HYBRID-value.

Although the total LF and WF amounts for food are not very different for most models, there are some quite marked differences between values for products or product groups (Fig. 2 and Supplementary Table 2). For the LF, animal product groups (dairy and meat) make up 61% of the total amount for PHYS, 67% for FABIO-mass/HYBRID-mass and 66% for FABIO-value/HYBRID-value, with substantial differences for single product groups (for example, beef 27.0 Mha for PHYS, 18.6 Mha for FABIO-mass/HYBRID-mass and 23.2 Mha for FABIO-value/HYBRID-value). For EXIOBASE, the distinction between different products and product groups is not very clear. As a result, the animal product groups sum up to lower values than in the other models, but part of these amounts are contained within the product group ‘Other food’ (which is definitely food) and ‘Undefined’ (where the distinction between food or non-food is not clear). EXIOBASE has much fewer separate food product items included. ‘Vegetables, fruit, nuts’ is, for example, a single product item in EXIOBASE, amounting to an LF of 20.9 Mha. In PHYS, FABIO and HYBRID, the group ‘Vegetables, fruit, nuts’ consists of many separate food items; the combined LF adds up to 9.4 Mha for PHYS and 7.3 Mha for FABIO and HYBRID, regardless of the allocation method (that is, mass or value). Especially for the animal products, the results from FABIO-mass/HYBRID-mass differ from those derived with FABIO-value/HYBRID-value. This difference can be explained by the fact that by-products used as feed often have a lower value per mass than the corresponding main product, thus receiving a smaller proportion of the environmental load when applying value-based rather than mass-based allocation. The observations for green WF for food are very similar to those of the LF.

The blue WF values of different product groups, such as cereals, are similar between models. Some product group footprints from FABIO-mass/HYBRID-mass differ slightly from the FABIO-value/HYBRID-value results, especially the animal products. The animal product groups in all models account for less of the total food amount than is the case for the LF and green WF. For PHYS the rate is 39%, for EXIOBASE-min 20%, for EXIOBASE-max 15%, for FABIO-mass/HYBRID-mass 41% and for FABIO-value/HYBRID-value also 41%. For the blue WF, the combined product groups vegetables, fruit and nuts account for a large proportion of the total value, that is, 28% for PHYS, 36% for EXIOBASE-min, 26% for EXIOBASE-max, 21% for FABIO-mass/HYBRID and 21% for FABIO-value/HYBRID-value. These proportions may be even higher for EXIOBASE-min and EXIOBASE-max because these products are partially included in the product groups ‘Other food’ and ‘Undefined’.

**Fig. 3:**
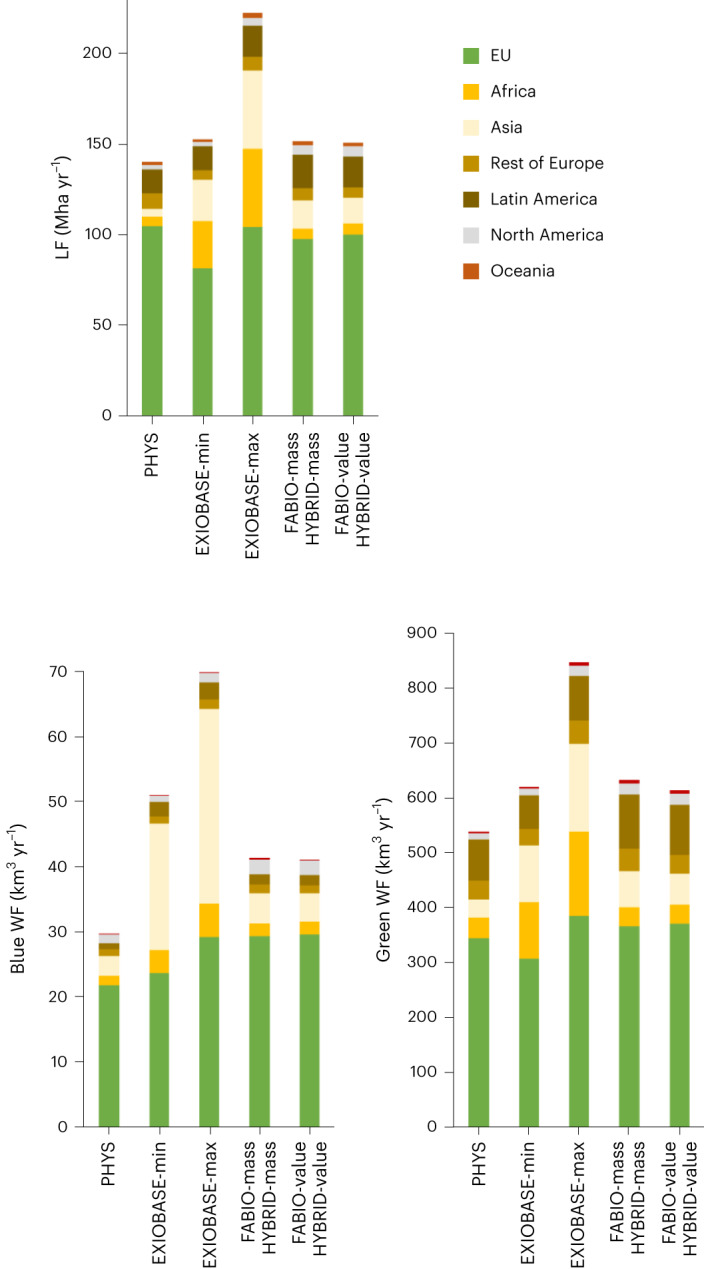
EU LF and WF according to the product region of origin for the seven model variations. LF, blue WF and green WF computed for products consumed in the EU according to their region of origin (by continent). Detailed product-level data are provided in Supplementary Tables 3–7.

The total LF and WF amounts for food are not very different between models, but we observed differences in amounts between models according to the origin of the products (Fig. 3). In PHYS, FABIO and HYBRID, the proportion of EU-produced food in the total footprints is relatively high (64–75% for the LF, 71–74% for the blue WF and 58–64% for the green WF). For the LF, the largest quantities of imported food come from Latin America (PHYS 13.4 Mha yr^−1^, FABIO-mass/HYBRID-mass 18.3 Mha yr^−1^ and FABIO-value/HYBRID-value 17.2 Mha yr^−1^), especially through (feed for) animal products. Also for the green WF, the largest quantities of imported food originate from Latin America (PHYS 74.5 km^3^ yr^−1^, FABIO-mass/HYBRID-mass 99.7 km^3^ yr^−1^ and FABIO-value/HYBRID-value 91.7 km^3^ yr^−1^) through meat and milk, but also coffee. Substantial amounts are also imported from Africa through cocoa and coffee, and from Asia through coffee. For the blue WF, however, the main imported proportion comes from Asia (PHYS 3.0 km^3^ yr^−1^, FABIO-mass/HYBRID-mass 4.6 km^3^ yr^−1^ and FABIO-value/HYBRID-value 4.2 km^3^ yr^−1^), largely through the import of pork and rice. In EXIOBASE, the proportion of EU-produced food in the total footprints is much smaller than in the other five models (47–53% for the LF, 42–47% for the blue WF and 46–50% for the green WF). For the LF and green WF, very large amounts of food are imported from Asia (22.9 Mha yr^−1^ for EXIOBASE-min and 42.9 Mha yr^−1^ for EXIOBASE-max for the LF, and 103.4 km^3^ yr^−1^ for EXIOBASE-min and 160.7 km^3^ yr^−1^ for EXIOBASE-max for the green WF) and Africa (26.0 Mha yr^−1^ for EXIOBASE-min and 43.2 Mha yr^−1^ for EXIOBASE-max for the LF, and 102.2 km^3^ yr^−1^ for EXIOBASE-min and 152.6 km^3^ yr^−1^ for EXIOBASE-max for the green WF). In particular, the EXIOBASE product categories ‘Food products nec (not elsewhere classified)’ and ‘Hotel and restaurant services’ represent large amounts. For the blue WF, Asia accounts for extremely high amounts of imported food (19.5 km^3^ yr^−1^ for EXIOBASE-min and 29.9 km^3^ yr^−1^ for EXIOBASE-max) compared with the total footprint. In particular, the EXIOBASE product categories ‘Vegetables, fruit, nuts’ (6.5 km^3^ yr^−1^), ‘Food products nec’ (5.4 km^3^ yr^−1^) and ‘Hotel and restaurant services’ (2.3 km^3^ yr^−1^) account for much of the imported food from Asia.

## Global LF

**Fig. 4:**
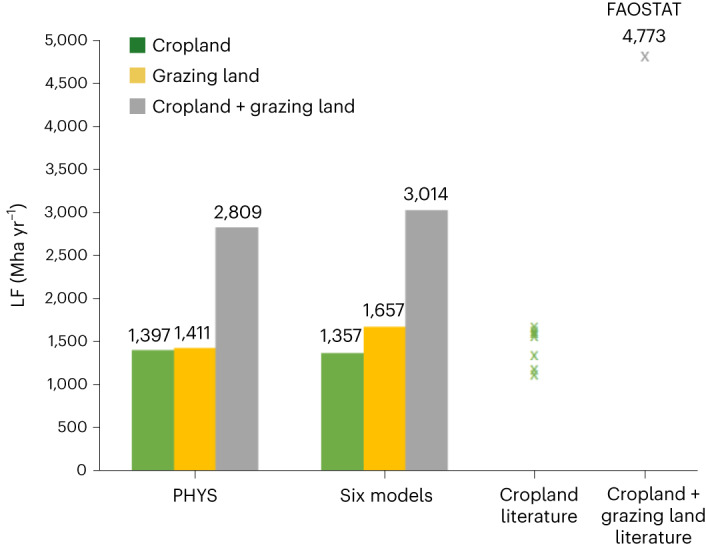
Global LF computed using the different models and comparison with the literature. Global LF for cropland and grazing land calculated in this study and comparison with literature values^13,26–29^. The total agricultural land use reported in FAOSTAT^13^ in 2012 amounts to 4,773 Mha, whereas our global LF estimate is 59–63% of this value. Our cropland statistics (1,357–1,397 Mha yr^−1^) are in the range of previous estimates (1,200–1,621 Mha)^26–29^. Our grazing land estimates (1,411–1,657 Mha yr^−1^) are considerably lower than those reported in FAOSTAT^13^.

We computed, for six out of the seven models (excluding PHYS), a global LF of 3,014 Mha yr^−1^ or 0.425 ha person^−1^ yr^−1^, of which 1,357 Mha yr^−1^ is from crop production and 1,657 Mha yr^−1^ from grazing (Fig. 4). For PHYS, we computed a global LF of 2,809 Mha yr^−1^, of which 1,397 Mha yr^−1^ is from crop production and 1,411 Mha yr^−1^ from grazing. The difference between PHYS and the other models comes from system boundaries, truncation and the inclusion/exclusion of certain products, such as camels (included in FABIO and EXIOBASE, but excluded in PHYS). The total agricultural land use (that is, cropland plus permanent meadows and pastures) reported in the Food and Agriculture Organization Statistics (FAOSTAT) database^13^ in 2012 amounts to 4,773 Mha, whereas our global LF estimate is 59–63% of this value. The difference is mainly explained by the grazing LF, as our cropland statistic is in the range of previous estimates (1,200–1,621 Mha)^26–29^, which includes the FAOSTAT cropland area of 1,544 Mha for the year 2012 (ref. ^13^).

## Discussion

### EU food LF and WF across models

Our multi-model analysis for the EU shows that it matters which accounting method is used. We observed general agreement in total LF and green WF amounts between six out of the seven models, whereas EXIOBASE-max resulted in substantially higher amounts. For the blue WF, PHYS computed lower and EXIOBASE higher total amounts than the other four models. The EXIOBASE-max amounts are higher as it includes product groups and services that potentially include food, thereby certainly overestimating the footprint values of food. However, we observed important differences between the models when evaluating product groups or product region of origin. Such differences are due to the fact that the models use different system boundaries and are built around different key assumptions^10^. For example, products in PHYS and FABIO are considered ‘consumed’ once they are converted into products that are not reported on by FAOSTAT (for example, palm oil that is used for the production of goods that are traded as cosmetics) as opposed to EXIOBASE and HYBRID, in which non-food supply chains are fully tracked until final consumption. In addition, the models apply different product classifications. While FABIO and EXIOBASE include trade in live animals, PHYS does not. Moreover, FABIO (hence also HYBRID) differentiates soybeans (or other oil seeds) that are traded and consumed as beans, vegetable oils or in the form of oil cakes, while in PHYS, soybeans and their derived products are currently converted into soybean equivalents and aggregated, and in EXIOBASE, they are part of several product groups (mainly ‘Oil seeds’, ‘Products of vegetable oils and fats’ and ‘Food products nec’).

A considerable difference between the models found in our analyses and caused by different underlying assumptions is how livestock feed is linked to livestock products. PHYS and FABIO use data from FAOSTAT on where feed crops are imported from and how much of them is used as feed. While FABIO splits these reported available feed amounts using the results from Bouwman et al.^30^ specifying feed requirements for 1970, 1995 and 2030, differentiating specific dietary requirements and feed compositions for cattle, buffaloes, pigs, poultry, sheep and goats in 17 world regions, PHYS applies a global weighting factor to distribute the feed across livestock products. This weighting factor is based on data from the United States, where livestock production systems are different from those in the EU (for example, cattle are often kept in feedlot systems in the United States, relying to a much larger degree on feed originating from cropland^31^). Thus, it likely overestimates feed use for beef within the EU, and consequently underestimates feed use for, for example, pork. Another example relates to the very high values for LF for ‘Meat other’ in FABIO, where it is not directly evident what food products consumed within the EU are linked to this category. This is most likely due to the fact that feed for leisure and sport horses is ultimately attributed to this final demand product in the absence of more precise data. Both models are presently being improved to include a better representation of livestock feed mixes, but the issue will remain a large factor in overall uncertainties due to existing data limitations and the high relevance of livestock feed use within the EU. In EXIOBASE, however, feed from the aggregated crop groups is assigned to animal husbandry according to their monetary value, which might underestimate actual quantities. Moreover, in contrast to PHYS and FABIO, it is no longer possible to clearly distinguish between animal and vegetable products in the footprints derived with EXIOBASE.

The key in LF and WF analyses is to use the same model or a multi-model assessment when performing scenario analysis, for example, of dietary behaviour or food loss and food waste reductions. Statements that mix the output of different models in their assessment should be avoided or at least interpreted with great care.

Many of the models used by researchers have become increasingly disaggregated with regards to products and countries. This is a positive evolution as, for example, the poor food product disaggregation in EXIOBASE leads to a wide range of uncertainty in footprint amounts between EXIOBASE-min and EXIOBASE-max. High food product disaggregation, including the identification of processed products, will reduce uncertainty in total food-related environmental footprints. This, in turn, enables more sophisticated scenario analysis. Recent research has shown that adding country resolution to formerly aggregated ‘ROW regions’ in EXIOBASE influences land-use accounting, rendering environmental footprint estimates more accurate^32^. Adding both country resolution and product/sectoral detail, aided by ever-increasing computing power, is thus of high value for environmental footprint studies. The challenge, however, is to update such detailed models on a regular basis to include recent years, for which research funding should be provided.

### Accounting for grazing land in LF analyses

The novel approach to accounting for grazing land in LF analyses presented here is aligned with the current standard in WF accounting, where only the green water associated with the grass eaten by livestock is counted, as opposed to the green water evapotranspirated from all lands grazed by livestock. Within the context of a footprint family assessment, harmonized approaches for different footprint calculations have much added value^6^. Our new global grazing LF of 1,411–1,657 Mha yr^−1^ represents an area hypothetically required if grazing land in a country were used at maximum intensity, given its current natural grazing land productivity. Using this approach for both footprint quantifications thus provides a standardization useful in footprint family assessments^6,7^.

By accounting for the intensity of grazing, not all grazing land is attributed to the LF of grazing. This accounting method provides lower LF amounts for low-intensity grazing systems, such as mountain regions or nomadic grazing regions in, for example, Eastern Africa or the Sahel. Here, livestock herds may roam vast areas, but actually eat only a fraction of the grass available.

In the current literature, accounting for grazing land in LF assessments is far from clear and leads to wide variations in computed amounts. Land-use science typically quantifies all areas assumed to be grazed by livestock in some way, with differentiations based on the intensity and frequency of grazing (Table 1). Large uncertainties relate to the extent of grazing land (38.8–61.9 Mkm^2^), of which 22.8–32.8 Mkm^2^ are permanent pastures and 6.1–39.1 Mkm^2^ are lands that are sporadically grazed by livestock but where grazing is not the dominant land use. In addition, a distinction relevant for climate/carbon models is typically made between grazing lands that have been converted from forests (associated carbon emissions have occurred) and lands with natural herbaceous cover (Table 1).

**Table 1:** Comparison of area-based estimates of global lands linked to livestock grazing

Category	Area (Mkm^2^)	Definition
**Grazing land**	48.0 (range 38.8–61.9) (ref. ^68^)	All land used for livestock grazing in any form
Permanent pastures (definition of FAOSTAT)	27.1 (range 22.8–32.8) (refs. ^13,19,24,26,68,69^)	Lands dominated by herbaceous forage crops (cultivated or natural), used predominantly for livestock grazing for 5 years or more
Intensive permanent pastures	2.6 (refs. ^68–70^)	Livestock density > 100 animals km^−2^
Extensive permanent pastures, on potential forest sites	8.7 (refs. ^68,70^)	Livestock density < 100 animals km^−2^ on lands potentially covered by forests
Extensive permanent pastures, on natural grasslands	15.8 (range 11.5–21.6) (ref. ^68^)	Livestock density < 100 animals km^−2^ on lands naturally covered by herbaceous vegetation
Non-forested, used land, multiple uses	20.1 (range 6.1–39.1) (ref. ^68^)	Lands that are sporadically grazed by livestock, but where grazing is not clearly the dominant land use
**Pastureland**, definition following the IUCN habitat scheme	2.1 (ref. ^33^)	Includes fertilized or re-seeded permanent grasslands, sometimes treated with selective herbicides, with very impoverished flora and fauna, and also secondary grasslands and wooded farmland
**Ecological footprint, grazing land component**	10.2 (ref. ^71^)	Expressed in global hectares (here converted to ‘global’ Mkm^2^) by standardizing for world average land productivity; the value in actual hectares based on the used equivalence factors^72^ would be 22.2 Mkm^2^
**This study: LF of livestock grazing**	14.1–16.6	Area hypothetically required if grazing land in a country were used at maximum intensity, given their current productivity (aligned with assumptions underlying WF accounting standards)

Another perspective is provided by conservation science, with the International Union for Conservation of Nature (IUCN) habitat category ‘Pastureland’ (Table 1). Here, the focus is typically on assessing whether land areas are still habitable by the original native species. A recent mapping of these habitat types^33^ gave a relatively low global estimate of the global ‘Pastureland’ category (2.1 Mkm^2^), highlighting that many land areas grazed by livestock still have potentially high biodiversity value.

In contrast, the ecological footprint standardizes also for land productivity and is thus reflective of the level of consumption, with the results given in global hectares (that is, hectares of standard global average land productivity, 10.2 Mkm^2^ for the grazing land component; Table 1). Therefore, comparison with estimates of actual area is not straightforward. Before applying the equivalence factor^33^ for the conversion to global hectares, the value would be 22.2 Mkm^2^.

When assigning the LF of livestock to products, an additional challenge relates to the fact that livestock are often not only kept for the products that they produce. For instance, PHYS does not assign grazing land use of buffaloes or camels to their meat production (while FABIO does), with the underlying assumption that their main use is often the provision of draught power and not meat. Similarly, cattle are often kept as insurance for extreme events in poorer contexts and not optimized for output. Assigning the entire LF of livestock to the product outputs might thus be an overestimation while underestimating the costs of other services that livestock provide.

### European Green Deal

The European Green Deal includes the Farm to Fork Strategy^3,4^, which aims to deliver a fair, healthy and environmentally friendly food system. This specifically includes the promotion of sustainable food consumption and facilitation of the shift to healthy, sustainable diets, as well as the reduction of food loss and waste. To quantify the environmental sustainability of the EU food sector, an assessment of its current LF and WF and target values is essential. We used a multi-model approach to analyse the situation for the year 2012, and the results call for care and consistency when selecting models and accounting methods to monitor progress and conduct scenario analysis.

We have also shown that models with high food product resolution show less uncertainty in total food footprints. The same is true for increasing country resolution in models^32^. Relevant major scenario analyses to decrease the food consumption LF and WF of the EU, also identified in the Farm to Fork Strategy, include shifting to a healthy sustainable diet^9,34,35^ and reducing food loss and waste along supply chains^36,37^. Detailed information regarding product and country of origin is essential in such analyses and resulting policy formulation

## Methods

We used FAOSTAT input data for the year 2012 as a basis for this study. This reference year was selected because it is the most recent year included in all models.

The population for the current EU27 in 2012 was 440,905,186 (ref. ^23^).

### LF accounting

To calculate the LF in PHYS, we first converted the trade flows obtained from the FAOSTAT database into primary crop equivalents and then accounted for re-exports. Then, we used additional data from the Food and Agriculture Organization commodity balances to calculate the flow of feed footprints embodied in the trade of animal products. Finally, we transformed the flows of primary products (in tonnes) into harvested area required for their production using annual and country-specific yield information of the producing country^11,38^.

For grazing land, we used a novel approach to translate the required amount of grass into a hypothetical area based on country-specific grassland yields. For this, we overlaid a spatially explicit pastureland layer^39^ with a layer of vegetation productivity^40^ and assumed that a maximum 75% of the aboveground net primary production (NPP) can be used by livestock; we then calculated the average grazing land productivity values per country. The productivity values derived in this way were then used to translate the grass feed intake estimates into an LF measure. In cases of very low land productivity, we cropped the grazing LF of a country at a maximum of 80% of the available land area. It is important to note that the resulting numbers are hypothetical in nature as grazing will often happen over larger areas and at lower intensities. However, this approach can be seen as a translation of the standard WF approach to LF accounting (see the Discussion section).

For clarification, we highlight in Table 2 that our calculation of the grazing land LF relies on the amount of grazed biomass and the productivity of potential grazing lands. Therefore, the same LF can be obtained in vastly different situations with vastly different actual grazing intensities (Case 1 versus Case 2). However, within a given area (for example, within a country), an increase in grazing intensity will lead to an increase in the grazing land LF.

**Table 2:** Calculation of grazing land LF

Variable	Case 1	Case 2	Used for our LF calculation
Area (km^2^)	200	25	
Land productivity^a^ (metric tonnes km^−2^)	20	40	Yes
Total grazable biomass (metric tonnes)	4,000	1,000	
Total grazed (metric tonnes)	500	1,000	Yes
**Grazing LF (km**^**2**^**)**	**25**	**25**	
Grazing intensity^b^ (%)	12.5	100	

Grazing land LF was calculated on the basis of the amount of grazed biomass and the productivity of potential grazing lands. ^a^Grazable biomass per area. ^b^Given as a percentage of the total grazable biomass.

We stress that the LF in this paper measures land use and not land-use change. It therefore does not provide information on the latter.

### Blue and green WF accounting

For the blue and green WF of crops, used as both food and feed, we used the international database of Mekonnen and Hoekstra^41,42^. The WF data in this database were analysed for the period 1996–2005. There is a slight time mismatch with the FAOSTAT input data for the year 2012, but up to this date, this database is the most comprehensive open access WF database available, which justifies its use. Methodologies to deal with the temporal dimension of crop WF exist^43^, but we did not apply them here.

The data on country-average water use (that is, actual evapotranspiration (ET)) from grazed pastures (m^3^ ha^−1^), averaged over 2000–2009, were obtained from Schyns et al.^24^. This dataset was generated by averaging gridded actual ET estimates (assumed to be fully green) as simulated with the LPJmL model using the daily grazing option under the livestock density that results in the highest grass yield^44^ over the estimated grazed area in a country. The grazed area for each 5 × 5 arc minute grid cell in a country was estimated as the area of permanent meadows and pastures^45^, after subtraction of the area under harvested fodder grasses^46^, for those grid cells where grazing livestock is mapped^47,48^. This provided a global green WF of grazing of 2,191 km^3^ yr^−1^, which is within the range of estimates from previous studies^24,25,49^.

Although some authors argue that accounting for both the LF and green WF results in double counting of environmental footprints (EFs)^50^, EF family assessment generally accounts for both^6,51,52^. Vanham et al.^6^ argue that the LF and green WF are both bound to land use, but they account for different resources: land and green water. They state that there is overlap but no double counting, in line with Hoekstra and Wiedmann^7^. The area of concern for the LF is limited land availability, expressed by Steffen et al.^53^ in a planetary boundary of land system change. For blue water, the area of concern is limited local blue water availability, accounting for environmental flows^2,54^, aggregated to a global blue water planetary boundary^53,55,56^. For green water, the area of concern is limited local green water availability, aggregated to a global green water planetary boundary^24^. The science on blue and green water planetary boundaries is currently evolving^57,58^, including with the recent publication of a green water planetary boundary^58^. The definition of the latter is quite different from the planetary boundary of land system change. Green and blue water are communicating vessels and their sum is limited by the available precipitation, which is essentially the resource that is being allocated to competitive uses^24^. Some measures in, for example, land-use management to increase green water availability might reduce beneficial blue water flows and vice versa. Some land-management measures affect both the LF and green WF, such as reforestation for ecosystem restoration. However, a land/soil-management practice such as mulching will have a substantial effect on the green WF^59^, but not on the LF. In the end, the aim is to make the different EFs of humanity sustainable, accounting for trade-offs and synergies^6^. It is thus justified to account for the LF, blue WF and green WF in a complementary manner. Their areas of concern differ and their solutions to achieve sustainability may also differ. In our assessment, we have also broken down the results into separate blue and green WF components, so that the reader can differentiate between the two.

Accounting for the LF, blue WF and green WF in a complementary manner is common practice in EF assessment^6^. However, in other frameworks, such as life cycle assessment (LCA), the green WF as a quantification of green water use is generally not used in combination with the LF or blue WF^60^. The usefulness of green water is largely questioned in the LCA^61^.

In our methodology, the WF and LF are attributed to final products and services, such as meat, milk or leather. The concept of ecosystem services (ES) is complementary to the EF family^6^. Only certain provisioning ES relate directly to or overlap with particular footprints^6^. Grazing provides for many non-provisioning ES, which we do not account for. Such ES include increasing plant species diversity and creating variation in plant structure, as cattle choose certain plants to eat over others, which is important for supporting a wide variety of wildlife species^62–64^.

### Trade models

We used a physical trade matrix model^18^ (PHYS) that accounts for 191 primary agricultural products and covers 223 countries. The model converts all products into primary crop equivalents. A detailed description can be found in the report by Schwarzmueller and Kastner^38^.

EXIOBASE covers 200 products and services, of which 19 are agricultural products, for 44 countries and 5 ROW regions. We used version 3.6 of the model, in which the time series in the period 1995–2011 from Stadler et al.^19^ has been updated with the year 2012, which is the restricting year for our multi-model analysis. The other models include more recent data, but EXIOBASE at the time of our analysis did not. For EXIOBASE-min, we attributed only the LF and WF of those product groups and services to ‘food’ that represent food for certain, whereas for EXIOBASE-max, we added product groups and services that potentially include food. We attributed the footprints of the remaining products to ‘non-food’. The selected food items for EXIOBASE-min and EXIOBASE-max are listed in Supplementary Table 1. A new EXIOBASE 3 variant expands regional coverage from 49 regions to 214 countries, but is defined by the authors as “still to be considered experimental”^32^. This is the reason we used the original EXIOBASE 3 version published in 2018.

The FABIO model^14^, a set of multi-regional supply, use and input–output tables in physical units that document the complex flows of agricultural and food products in the global economy, assembles FAOSTAT statistics reporting crop production, trade and use in physical units, supplemented by data on technical and metabolic conversion efficiencies, into a consistent, balanced MRIO framework. FABIO v1.1 covers 125 agriculture and food products for 191 countries and 1 ROW region from 1986 to 2013 (ref. ^65^).

HYBRID is a hybrid model that integrates both FABIO and EXIOBASE into a mixed-unit MRIO model covering agri-food supply chains in physical units and non-food and service supply chains in monetary units^20^.

Other MRIO models exist, such as EORA^66^ and GTAP (Global Trade Analysis Project)^67^, but these were not included in our study.

## Supplementary information

Supplementary Tables Supplementary Tables 1–7.

## Data Availability

FAOSTAT input data for the year 2012 are freely available. The data that support the findings of this study are available within the paper, its Supplementary Information and from the corresponding author upon reasonable request.

## Code availability

The codes used in this paper can be requested by contacting the authors.
